# Regional economic resilience and sustainable development: Does industrial diversity matter?

**DOI:** 10.1371/journal.pone.0342488

**Published:** 2026-02-13

**Authors:** Shangzhi Mu

**Affiliations:** Party School of Liaoning, Provincial Party committee, Shenyang, Liaoning, China; Southwestern University of Finance and Economics, CHINA

## Abstract

Regional sustainable development is a fundamental pathway toward achieving long-term economic stability, social equity, and ecological balance. Particularly in the face of unexpected shocks and systemic risks, economic resilience plays a critical role in ensuring the attainment of sustainability goals. Drawing on endogenous growth theory and dynamic capabilities theory, this study focuses on 31 provincial-level administrative divisions in mainland China as the target regions, utilizing panel data from 2004 to 2023 to empirically examine the relationships. The baseline regression results indicate a significant positive effect of economic resilience on sustainable development. Moderation analysis further reveals that industrial diversification, human capital, and environmental regulation significantly enhance the positive impact of resilience. Mediation tests show that economic resilience promotes sustainable development indirectly by improving the level of marketization. Heterogeneity analysis confirms that the impact of economic resilience on sustainable development varies significantly across regions. The findings of this study provide a scientific basis for governments to formulate more effective risk-mitigation policies and for firms to identify more stable regions for investment.

## 1. Introduction

Amidst the impact of black swan events and technological transformations on the global economic system, the stability and sustainability of regional development are undergoing unprecedented challenges. The diffusion of digital technologies is reshaping the traditional production structures, while constraints associated with the green transition are forcing economies to reassess the trade-off between efficiency and ecological costs. At the same time, geopolitical fluctuations and risks of supply chain disruptions are exacerbating uncertainties in growth pathways [1]. Compound crises have revealed that economies overly reliant on a single industry are prone to fall into a recovery growth trap following external shocks—that is, after a short-term rebound, they struggle to overcome structural growth bottlenecks due to insufficient innovation momentum [2]. Significant disparities have also emerged in the economic development of different regions. Those dependent on a monolithic industrial structure often exhibit greater vulnerability when confronted with external shocks, frequently experiencing severe economic fluctuations or even long-term recessions. In contrast, regions characterized by diversified industrial structures generally demonstrate stronger adaptive and recovery capacities. In recent years, with the deepening promotion of the philosophy of sustainable development, how to achieve coordinated unification of stable economic growth, environmental protection, and social progress has become a crucial issue for regional development strategies. Industrial structure diversity, as an important feature of regional economic systems, is being recognized and reevaluated. Within the macro context of global value chain restructuring and accelerated low-carbon transition, the degree of industrial diversification directly influences a region’s ability to cope with uncertainty and is also integral to the effectiveness of nurturing emerging industries and constructing innovation ecosystems. Therefore, conducting an in-depth exploration of the mechanism through which industrial structure diversity influences the relationship between regional economic resilience and sustainable development holds significant theoretical and practical importance for formulating effective regional development policies. This study targets China’s 31 provincial-level administrative divisions, leveraging their diverse economic landscapes to investigate these dynamics.

Existing literature has extensively examined relevant variables; however, several limitations persist despite this growing body of research. First, mainstream indicators—such as GDP rebound rates—tend to overlook the dynamic adaptability of economic resilience in response to prolonged and compounding shocks. These measures are also ill-suited to capture regional heterogeneity in developing countries. Moreover, the mechanisms through which economic resilience fosters sustainable development remain unclear, particularly due to the lack of quantitative evidence regarding the marketization transmission effect and the moderating role of industrial diversity. Finally, prevailing theoretical frameworks developed in advanced economies often neglect the interplay between path dependency rigidity and industrial structure in developing contexts. In light of these gaps, this study seeks to address the following research questions: (1) How can a comprehensive and integrated method for measuring economic resilience be constructed? (2) Does industrial structure diversity play a positive role? (3) Does economic resilience drive sustainable development through market-based allocation?

This paper investigates the mechanisms by which regional economic resilience contributes to sustainable development and explores the role of industrial structural diversity in this relationship. The findings suggest that enhanced economic resilience facilitates sustainable development within a region. Furthermore, industrial structural diversity, human capital and environmental regulation significantly amplify this positive impact. The study also uncovers the transmission pathway through which economic resilience operates—by improving the level of marketization, resilience effectively promotes sustainability. Additionally, regional development characteristics, including endogenous capacity and external connectivity, are shown to influence the effectiveness of resilience in diverse ways.

The theoretical contributions of this study are threefold. First, it constructs a “Resilience-Marketization-Sustainability” theoretical framework, elucidating the intrinsic transmission pathways through which regional resilience influences development, building upon Martin’s (2012) work on regional resilience during recessions [3] by integrating marketization as a mediator in developing economies. Second, it innovatively proposes a synergistic moderating model integrating industrial structure and human capital, thereby transcending the limitations of single-factor analysis. This advances Boschma’s (2015) evolutionary perspective on regional resilience [4] by incorporating multi-factor interactions that address path dependency in diversified structures. Third, by differentiating the heterogeneous effects of endogenous dynamics and external connectivity, it establishes a regionally differentiated resilience evaluation system. This contributes to Sensier et al.’s (2016) measurement of European regional resilience [5] by adapting it to heterogeneous developing contexts like China, emphasizing endogenous and external factors.

## 2. Literature review

Research related to this study primarily focuses on the measurement of regional economic resilience [5,6], and the linkage mechanisms between economic resilience and sustainable development [7]. The existing literature is reviewed below from three perspectives.

First, concerning the measurement methods of regional economic resilience, current approaches can be classified into two categories: The first category is relies on economic recovery indicators, such as the rebound speed of employment rates or GDP following a shock. Sensier, Gillian [5] proposed employment recovery elasticity as a core metric based on their analysis of recovery cycles in European regions after economies downturns. However, this method overlooks the impact of structural transformation. The second category employs composite indices, Lin, Peng [6] constructed an indicator system encompassing economic diversity, innovation capacity, and institutional quality, yet the subjective determination of weights within this system remains controversial [1].

Second, research on the interaction between economic resilience and sustainable development indicate that economic resilience serves is a crucial safeguard for sustainable development. From a macro perspective, Martin [3] examined how economic resilience aids in understanding regional responses to major recessionary shocks.

From an industrial perspective, Liu, Wu [7] argued that industrial clusters contribute to reducing carbon emissions. Finally, regarding the moderating role of industrial diversity, the buffering effect hypothesis posits that a diversified industrial structure enhance shock resistance by dispersing risks [2]. Conversely, the innovation-catalysis hypothesis emphasizes that related diversity promotes green innovation through technological spillovers, thereby enhancing sustainability [8]. However, some studies present contrasting views, suggesting that excessive diversification may lead to resource dispersion [9]. These divergent perspectives underscore the need for empirical moderation analyses, as pursued in this study, to reconcile buffering effects with potential inefficiencies in resource allocation.

Despite these substantial contributions, several aspects warrant further exploration. First, existing research predominantly examines factors influencing economic resilience, largely neglecting its impact on comprehensive regional development. Second, most studies rely on static cross-sectional data, failing to capture the path-dependent characteristics inherent in the evolution of regional resilience.

### 2.1. Theoretical foundation and research hypotheses

#### 2.1.1. Endogenous growth theory.

Regional economic resilience refers to the structural and dynamic capacity of a regional economy to withstand external shocks, maintain core functions, recover stable states, and achieve adaptive transformation. It represents a critical dimension in promoting high-quality regional development. Sustainable development emphasizes meeting contemporary needs without compromising the ability of future generations to meet their own needs. Its realization strongly depends on innovation-driven growth, efficient resource allocation, and effective institutional coordination as intrinsic growth pathways. Endogenous growth theory provides a solid micro-mechanism and macro-theoretical foundation for systematically understanding the impact of regional economic resilience on sustainable development capacity, as well as the key moderating role of human capital within this relationship.

Unlike neoclassical growth models that emphasize exogenous technology and factor accumulation, endogenous growth theory focuses on explaining the fundamental drivers of long-term sustainable development through endogenous factors within the economic system, such as knowledge creation, technological innovation, R&D activities, and human capital accumulation. Within this framework, technological progress is no longer exogenously determined but is endogenously shaped by R&D investments, knowledge stocks, learning effects, and policy incentives within the economic system. The enhancement of regional economic resilience relies precisely on such endogenous resource and capability foundations, rather than solely on external resource inputs. First, high-quality knowledge innovation capacity serves as the central engine for regional learning, adaptation, and transformation potential. It directly drives technology absorption and independent innovation while continuously improving total factor productivity through knowledge spillovers. Second, the connectivity and synergy of industrial structure enhance risk dispersion capabilities and improve the efficiency of resource reallocation, significantly strengthening the system’s robustness and recovery capacity in response to external disturbances. Furthermore, institutional efficiency and governance capacity, as important contextual conditions, directly influence knowledge dissemination mechanisms and innovation incentives, serving as guarantees for translating resilient capabilities into tangible developmental outcomes. Endogenous resources, through continuous accumulation, transformation, and restructuring effects, lay a solid foundation for sustainable development paths that balance efficiency improvements, social inclusion, and ecological sustainability.

Based on the above analysis, this study proposes Hypothesis 1:

*Hypothesis 1: Regional economic resilience significantly promotes sustainable development.*

This hypothesis builds on the endogenous growth models of Romer (1990) and Lucas (1988), which emphasize knowledge accumulation and human capital as drivers of sustained growth, by extending them to incorporate resilience as an endogenous factor in regional sustainability.

Simultaneously, endogenous growth theory profoundly reveals the central role of human capital in economic growth and development quality. It is not only an indispensable factor of production but also a core active carrier and spillover hub for knowledge creation, technology absorption, and application diffusion. Based on this, this study further argues that the level and structure of existing regional human capital are key factors that significantly moderate the intensity and efficiency of the impact of regional economic resilience on sustainable development. Regions with high human capital typically possess stronger talent reserves, denser knowledge stocks, and more robust education innovation networks, which significantly enhance the learning, adaptation, and transformation capacities of regions facing shocks. When confronted with external shocks, such regions can more effectively leverage their industrial diversity and institutional flexibility to quickly identify technological opportunities, absorb green innovation knowledge, and apply more sustainable development models, thereby translating resilient capacities into substantive sustainable development performance.

Moreover, human capital-intensive regions often feature more active innovation collaboration networks, more efficient knowledge exchange mechanisms, and a stronger entrepreneurial atmosphere. This greatly enhances the efficacy and speed with which regions, following shocks, can leverage their economic resilience foundations to direct physical capital, data factor, and policy resources toward green technologies, low-carbon industries, and inclusive social sectors. In other words, the innovation spillover and factor upgrading effects driven by human capital can transform the short-term stability and recovery effects brought by economic resilience into significant and long-term ecological benefits, social welfare improvements, and industrial structure advancement, thereby effectively amplifying the positive impact of resilience on sustainable development.

Thus, this study proposes the following moderating effect hypothesis:

*Hypothesis 2: Human capital significantly enhances the positive effect of regional economic resilience on sustainable development.*

#### 2.1.2. Dynamic capabilities theory.

According to the dynamic capabilities theory, organizations can establish and sustain competitive advantages by identifying environmental changes, seizing transformation opportunities, and reconfiguring their resource base. Regional economic resilience, as a spatial manifestation of this theory, reflects a region’s comprehensive capacity to withstand shocks, adapt to changes, and achieve structural transformation. Industrial structure diversity plays a pivotal role in activating dynamic capabilities and enhancing the facilitative effect of economic resilience on sustainable development. The mechanism operates primarily through three dimensions: strengthening environmental sensing, improving opportunity capture, and reinforcing resource reconfiguration. A diversified industrial system, leveraging its cross-sectoral information networks and knowledge exchange nodes, broadens and deepens the region’s ability to perceive and respond to external changes. Moreover, industrial diversity provides regional economies with resource buffers and alternative development pathways, enabling swift identification and activation of new growth drivers when specific industries or technological trajectories are disrupted. Furthermore, the intersection and integration of knowledge, skills, and technologies across different industries create fertile ground for green technology innovation and the emergence of sustainable business models. Thereby, industrial diversity endows economic resilience with a more proactive developmental orientation, upgrading it from a defensive capacity against shocks to a positive driver of regional sustainable development, significantly amplifying its positive influence. Based on this analysis, the following hypothesis is proposed:

*Hypothesis 3: Industrial diversity positively moderates the relationship between regional economic resilience and sustainable development.*

This extends Teece et al.‘s (1997) dynamic capabilities framework, which focuses on firm-level adaptation, to the regional level by highlighting how industrial diversity enables sensing, seizing, and reconfiguring opportunities for sustainability.

As an external institutional factor, environmental regulation further strengthens the promoting effect of economic resilience on regional sustainable development capacity. The core mechanism lies in the fact that environmental regulations, by establishing clear constraints and incentives, not only reshape corporate behavioral decisions but also profoundly influence the direction of regional resource flows and technological innovation, thereby triggering systemic and structural adaptation and transformation. Economic resilience provides the foundational capacity for regions to cope with external pressures, while environmental regulations impose compulsory momentum for green transition, channeling such capacity toward the goals of sustainable development. Specifically, environmental regulation enhances the positive effect of economic resilience through two mechanisms. First, by raising environmental compliance costs and emission standards, it pushes enterprises to reallocate resources toward green technological innovation and efficiency improvements, accelerating the phase-out of polluting and inefficient production capacities, and facilitating a transition toward a greener and more advanced economic structure. Regions with higher economic resilience can adapt more effectively to such regulatory pressures, turning constraints into opportunities for industrial upgrading and technological innovation. Second, environmental regulation reduces uncertainties during transition through stable policy signals and clear green development guidance, directing long-term capital into sectors such as energy conservation, environmental protection, new energy, and ecological governance, thereby fostering the development of green financial systems and relevant human capital. Regions with strong economic resilience are better able to respond to these institutional signals, integrate market and social resources, and form a synergistic development path that integrates environmental and growth objectives. Thus, the following hypothesis is proposed:

*Hypothesis 4: Environmental regulation enhances the positive impact of economic resilience on regional sustainable development.*

As a system-level dynamic capability, the sustainable development efficacy of regional economic resilience relies on effective institutional mediation, with the level of marketization serving as a key mechanism. Regions with higher economic resilience are more adept at identifying institutional deficiencies within the market mechanism and initiating adaptive adjustments, such as strengthening property rights protection, improving contract enforcement, and optimizing factor market allocation, thereby systematically enhancing the degree of regional marketization. The improvement of marketization, as an important mediator between economic resilience and sustainable development, operates primarily through two channels: reconstructing resource allocation efficiency and incentivizing innovation investment. On one hand, enhanced factor mobility significantly reduces resource misallocation, facilitating the transfer and aggregation of capital, labor, and technology from inefficient and polluting sectors to efficient and green sectors. On the other hand, well-defined property rights, effective contract enforcement, and predictable policy environments substantially reduce the institutional risks associated with green technological innovation and long-term environmental investment, strengthening the willingness and capacity of market entities to participate in the green transition. Therefore, marketization is not only an important manifestation of economic resilience but also a crucial transmission mechanism that transforms resilient advantages into improved economic efficiency, socially inclusive development, and reduced environmental pressure. Based on the above discussion, the following hypothesis is proposed:

*Hypothesis 5: Economic resilience promotes regional sustainable development by enhancing marketization level.*

**Fig 1:**
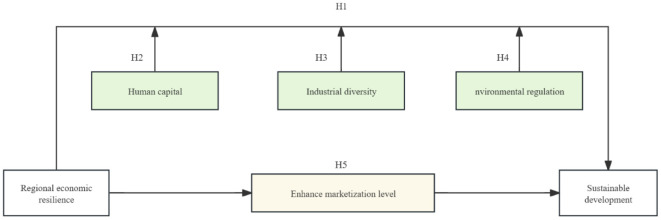
Research framework.

In conclusion, the research framework of this paper is shown in Fig 1.

## 3. Research design

### 3.1. Data sources and processing

This study employs provincial-level panel data from Chinese spaning 2004–2023 for empirical analysis. After removing observations with missing values and winsorizing continuous variables at the 1st and 99th percentiles, the final sample comprises 544 observations. The panel covers 31 provincial-level administrative divisions in mainland China over 20 years (2004–2023), yielding an initial unbalanced panel of up to 620 potential observations (31 provinces × 20 years); the reduction to 544 observations accounts for missing data in variables such as economic indicators or environmental metrics across certain province-year combinations.

### 3.2. Variable definition and descriptive statistics

#### 3.2.1. Dependent variable.

Sustainable development (*SUS*) is measured following the approach of [10], by constructing a comprehensive evaluation system across three dimensions: economic, social, and environmental. The entropy method is used for quantitative analysis. Specifically, the economic dimension is evaluated through three core indicators: development level, economic structure, and market vitality. The social dimension measures stability through key elements such as economic security and social protection mechanisms. The environmental dimension focuses on core indicators like ecological quality, energy efficiency, and pollution control. Through the combined computation of these multidimensional indicators, a comprehensive system for evaluating regional sustainable development capacity is developed. A higher *SUS* value indicates a stronger capacity for sustainable development in the region. The detailed indicator system is presented in Table A.1 in S1 Appendix. Based on this system, the variable *SUS1* was derived using principal component analysis for subsequent robustness checks.

#### 3.2.2. Independent variable.

Economic resilience (*Economy_resil1*) is typically measured in two main ways in existing research. The first method involves calculating the changes in unemployment rates or GDP growth, while the second method constructs a composite evaluation system. Regional economic resilience is a relative evolutionary indicator of a region’s economic system, and using a single indicator for measurement fails to accurately capture the full meaning of regional economic resilience, making it less convincing. This study constructs an indicator system to measure economic resilience. Following the approach of Ji and Huang [11], the indicators are selected based on three dimensions: resistance and recovery capacity, adaptability and adjustment capacity, and innovation and evolution capacity. The indicators are calculated using the entropy method for different sub-indicators, resulting in *Economy_resil1* and *Economy_resil2*. *Economy_resil2* is used for subsequent robustness tests. The sub-indicators for *Economy_resil1* are detailed in Table A.2 in S1 Appendix, while those for Economy_resil2 are presented in Table A.3 in S1 Appendix. *Economy_resil1* places greater emphasis on integrated capabilities encompassing resistance and recovery, adaptation and adjustment, as well as innovation and evolution. *Economy_resil2*, meanwhile, extends beyond this foundation by incorporating additional indicators, such as fiscal revenue and expenditure, education and technology investment, and industrial structure advancement, that reflect regional long-term adaptive capacity and developmental potential.

#### 3.2.3. Moderating variables.

Human Capital (*Human*), Human capital is measured by the relative ratio of the number of enrolled students in higher education institutions to the total population in a region. This indicator effectively reflects the region’s reserve of knowledge-based labor and its development potential. A higher ratio signifies that the region possesses a more abundant human resource endowment and stronger innovation potential, thus providing essential human capital support for transforming economic resilience into sustainable development momentum.

Industrial diversity (*DIV*), for measuring *DIV*, this study adopts the information entropy-based index construction method following the approaches of Frenken, Van Oort [12] and Duarte and Carvalho [13]. The specific calculation formula is as follows:


$${DIV}_{it}=\sum {\mathrm{\backslash nolimits}}_{i=\text{1}}^{n}P_{i}\;\text{ln}\left(\frac{\text{1}}{P_{i}}\right)$$ (1)

Where ${DIV}_{it}$ is the industrial diversity index, and $P_{i}$ represents the employment share of a specific industry within the province. This index utilizes employment shares as weights to reflect the degree of industrial diversification through entropy calculation. A higher index value indicates a more diversified industrial structure in the province.

Regarding environmental regulation (*Regulation*), this study measures the intensity of environmental regulation through the ratio of completed industrial pollution control investment to industrial added value across regions, following the methodologies of Yan, Yu [14] and Tang, Chen [15]. An increase in this ratio indicates that more economic resources are being allocated to reduce pollution per unit of industrial output, reflecting stronger enforcement of environmental regulations in the region, greater government emphasis on environmental protection, more stringent constraints on pollution behavior.

#### 3.2.4. Mediating variable.

Marketization level (*Market*), Based on the research by Fu, Luo [16], the marketization level is measured using the Fan Gang Marketization Index. This index provides a comprehensive evaluation of regional market development levels across five aspects: the development of factor markets, the relationship between government and markets, the development of market intermediaries, and the legal and institutional environment.

#### 3.2.5. Control variables.

Based on the approach of Ullah, Nobanee [17], the following indicators are selected as control variables and included in the regression model: fiscal support intensity (*FS*), urban-rural income gap (*Gap*), consumption level (*CPI*), government intervention level (*GI*), innovation level (*IL*), industrial upgrading (*ISU*), and energy structure (*ES*). The calculation methods for these variables are detailed in Table 1.

**Table 1:** Variable definitions.

Type	Variable	Symbol	Definition
Dependent variable	Sustainable development	*SUS*	Composite score calculated using the entropy method based on indicators from economic, social, and environmental dimensions.
Independent variable	Regional economic resilience	*Economy_resil1*	Indicators selected from resistance and recovery capacity, adaptability and adjustment capacity, and innovation and evolution capacity, calculated using the entropy method.
Moderating variables	Human capital	*Human*	Number of university students/total population
	Industrial diversity	*DIV*	See Equation (1).
	Environmental regulation	*Regulation*	The ratio of completed investment in industrial pollution control to industrial added value
Mediating variable	Marketization level	*Market*	According to Fu, Luo [16], the index of marketization level was calculated
Control variables	Fiscal support intensity	*FS*	Fiscal budget expenditure/GDP
	Urban-rural income gap	*Gap*	Urban residents’ disposable income/rural residents’ disposable income
	Consumer price index	*CPI*	Consumer price index.
	Government intervention	*GI*	Public budget expenditure/GDP
	Innovation level	*IL*	Ln of the number of patent applications
	Industrial upgrading	*ISU*	(First industry output/GDP) + 2×(Second industry output/GDP) + 3×(Third industry output/GDP)
	Energy structure	*ES*	Regional electricity consumption/ Total electricity consumption

#### 3.2.6. Descriptive statistics.

This study conducts statistical analysis on the distribution characteristics of *SUS*, *Economy_resil1*, and *Economy_resil2*. The distribution of *SUS* is relatively concentrated, with its mean significantly exceeding the median, and no extreme outliers are observed. *Economy_resil1* exhibits a central tendency similar to *SUS* but demonstrates significantly greater dispersion. *Economy_resil2* displays a distinct bimodal distribution tendency, with the majority of observations concentrated in the lower-value range (Table 2).

**Table 2:** Summary statistics.

VarName	Obs	Mean	SD	Min	Median	Max
*SUS*	544	0.3225	0.1605	0.0827	0.2894	0.7658
*SUS1*	544	0.0063	1.2892	−1.9332	−0.2175	4.3543
*Economy_resil1*	544	0.2211	0.1884	0.0345	0.1468	0.7739
*Economy_resil2*	544	0.2790	0.1942	0.0430	0.2144	0.9444
*DIV*	544	1.0161	0.0949	0.5569	1.0441	1.0985
*Human*	544	0.0182	0.0065	0.0058	0.0179	0.0358
*Regulation*	544	0.0041	0.0035	0.0002	0.0031	0.0190
*Market*	544	7.5034	1.9848	2.0230	7.4940	11.5440
*FS*	544	0.2448	0.1631	0.0924	0.2119	1.2748
*Gap*	544	3.4782	3.5655	1.8551	2.6960	26.3208
*CPI*	544	102.5967	1.6862	98.5000	102.3000	107.6000
*GI*	544	0.2368	0.1496	0.0877	0.2071	1.1289
*IL*	544	8.8214	1.7203	4.5218	8.9008	12.1455
*ISU*	544	2.3269	0.1433	1.9875	2.3140	2.7973
*ES*	544	0.0330	0.0234	0.0009	0.0256	0.0986

### 3.3. Model construction

This study establishes the following fixed-effects model to test the hypotheses proposed in this paper. The fixed-effects model is chosen for the following reasons: First, significant differences exist across China’s provinces in terms of resource endowment, policy implementation, and other factors. Fixed effects effectively control for these region-specific characteristics that do not change over time, thus avoiding omitted variable bias. Second, the study focuses on the dynamic impact of regional economic resilience on sustainable development within each province, making the fixed-effects model more suitable for capturing the temporal changes in this relationship. Third, the Hausman test results support the superiority of the fixed-effects model over the random-effects model. This model specification aligns well with the theoretical framework of regional dynamic capability evolution in this study.


$$\begin{array}{c} {SUS}_{it}=\alpha_{\text{0}}+\alpha_{\text{1}}{Economy\_resil\text{1}}_{it}+\sum {\mathrm{\backslash nolimits}}_{i=\text{1}}^{n}\omega_{i}X_{it}+\delta_{i}+\mu_{t}+\varepsilon_{it} \end{array}$$ (2)

$$\begin{array}{c} {SUS}_{it}=\beta_{\text{0}}+\beta_{\text{1}}{Economy\_resil\text{1}}_{it}+\beta_{\text{2}}M_{it}{+\beta }_{\text{3}}{Economy\_resil\text{1}}_{it}\times M_{it}+\sum {\mathrm{\backslash nolimits}}_{i=\text{1}}^{n}\omega_{i}X_{it}+\delta_{i}+\mu_{t}+\varepsilon_{it} \end{array}$$ (3)

$$\begin{array}{c} Z_{it}=\theta_{\text{0}}+\theta_{\text{1}}{Economy\_resil\text{1}}_{it}+\sum {\mathrm{\backslash nolimits}}_{i=\text{1}}^{n}\omega_{i}X_{it}+\delta_{i}+\mu_{t}+\varepsilon_{it} \end{array}$$ (4)

$$\begin{array}{c} {SUS}_{it}=\varphi_{\text{0}}+\varphi_{\text{1}}{Economy\_resil\text{1}}_{it}+\varphi_{\text{2}}Z_{it}+\sum {\mathrm{\backslash nolimits}}_{i=\text{1}}^{n}\omega_{i}X_{it}+\delta_{i}+\mu_{t}+\varepsilon_{it} \end{array}$$ (5)

In Equation (2), the model serves as the baseline regression model, while Equation (3) represents the moderating effect model, where $M_{it}$ denotes the moderating variable. Equations (4) and (5) represent the mediation effect models, with $Z_{it}$ as the mediating variable. Here, i represents the province, and t represents the year. ${SUS}_{it}$ refers to sustainable development capacity, ${Economy\_resil\text{1}}_{it}$ represents regional economic resilience, and $X_{it}$ are the control variables in the model. $\delta_{i}$ denotes province fixed effects, $\mu_{t}$ represents time fixed effects, and $\varepsilon_{it}$ is the error term.

## 4. Empirical analysis

### 4.1. Baseline regression

Table 3 presents the regression results for Equation (2). The preliminary results in Column (1) show that the coefficient of *Economy_resil1* is significantly positive at the 1% level. This finding directly validates the core proposition of endogenous growth theory: the endogenous resources and capabilities underpinning resilience serve as a key engine driving sustainable development. Column (2) further incorporates critical control variables affecting *SUS*. Although the coefficient of *Economy_resil1* exhibits an expected and reasonable attenuation, it remains highly significant at the 1% level with a stable direction. This reinforces the theoretical expectation that the positive effect of economic resilience on sustainable development does not stem from omitted variable bias. Instead, it originates from resilience’s endogenous growth-driving attributes, which independently and persistently enhance regional sustainability levels. Furthermore, the coefficient estimate indicates that a one-standard-deviation increase in *Economy_resil1* leads to a 1.2218 standard-deviation increase in *SUS*. To address potential model specification errors, Column (3) employs a random effects model, while Column (4) adopts a dynamic panel system GMM approach for comparison. Across both models, the significantly positive impact of *Economy_resil1* remains highly consistent with the baseline fixed effects model. This methodologically confirms the reliability of Hypothesis 1. These consistent results across models align with the endogenous growth theory’s emphasis on resilience as an internal driver, further supported by the control variables’ stability.

**Table 3:** Baseline regression.

	(1)	(2)	(3)	(4)
	*SUS*	*SUS*	*SUS*	*SUS*
*Economy_resil1*	1.0804***	1.0409***	0.7514***	0.1879***
	(22.2012)	(17.6331)	(19.8139)	(2.7475)
*FS*		0.0777	0.1001	−0.1108*
		(1.2966)	(1.6378)	(−1.6736)
*Gap*		−0.0021	−0.0015	−0.0007***
		(−1.4808)	(−1.3947)	(−2.9691)
*CPI*		−0.0057***	−0.0048***	0.0021**
		(−3.4083)	(−2.7607)	(2.0931)
*GI*		−0.0923	−0.1736**	0.1151
		(−1.3570)	(−2.5429)	(1.3860)
*IL*		0.0018	0.0083**	0.0076***
		(0.4837)	(2.4608)	(3.5901)
*ISU*		0.0057	−0.0126	−0.0524***
		(0.3471)	(−0.7530)	(−2.8727)
*ES*		0.2352	0.6185***	0.3995**
		(0.7327)	(2.6761)	(2.4689)
*L.SUS*				0.6858***
				(6.6218)
_cons	0.0761***	0.6571***	0.6119***	−0.1064
	(6.9435)	(3.6665)	(3.2947)	(−0.8020)
Year FE	YES	YES	YES	YES
Province FE	YES	YES	YES	YES
*N*	544	544	544	512
adj. *R*^2^	0.5770	0.5865		
AR (1)				0.0009
AR (2)				0.6245
Hansan				0.4068

Note: *** indicates p < 0.01, ** indicates p < 0.05, * indicates p < 0.1. The values in parentheses are t-statistics. FE stands for fixed effects. The same notation applies to the tables below.

### 4.2. Mechanism analysis

#### 4.2.1. Moderating effects.

Table 4 reports the regression results for Equation (3), the moderating effects model. The key finding is that the coefficient of the interaction term *Economy_resil1* × *Human* is significantly positive. This provides strong empirical support for Hypothesis 2, that is, Human positively moderates the promoting effect of *Economy_resil1* on *SUS*. Specifically, in regions with high human capital, the positive impact of economic resilience enhancement on sustainable development is significantly stronger than in regions with low human capital. This result aligns with endogenous growth theory expectations. Higher human capital levels signify that regions possess stronger knowledge absorption capabilities, technological innovation conversion efficiency, and adaptive labor reserves. When regions face external shocks or pursue transformation, abundant human capital can more effectively translate the risk-resistant potential embedded in economic resilience into substantive sustainable development outcomes.

**Table 4:** Moderating effects.

	(1)	(2)	(3)	(4)
	*SUS*	*SUS*	*SUS*	*SUS*
*Economy_resil1*	1.0409***	0.8867***	0.7248***	1.0061***
	(17.6331)	(13.7055)	(6.6140)	(17.5832)
*Human*		5.0012***		
		(9.0720)		
*Economy_resil1* × *Human*		3.1467**		
		(2.0892)		
*DIV*			0.1624***	
			(4.3924)	
*Economy_resil1* × *DIV*			0.2622***	
			(2.6495)	
*Regulation*				−2.8202***
				(−5.3648)
*Economy_resil1* × *Regulation*				17.6342***
				(6.0944)
_cons	0.6571***	0.1966	0.0216	0.5772***
	(3.6665)	(1.2278)	(0.1221)	(3.2972)
Control	YES	YES	YES	YES
Year FE	YES	YES	YES	YES
Province FE	YES	YES	YES	YES
*N*	544	544	544	544
adj. *R*^2^	0.5865	0.6891	0.6556	0.6169

Second, the interaction term *Economy_resil1* × *DIV* exhibits a significantly positive effect. It is indicated that *DIV* has amplified the positive effect of *Economy_resil1* on *SUS*, and Hypothesis 3 has been verified. This moderating effect stems from *DIV*’s systemic activation of regional dynamic capabilities: cross-industry information networks enhance the breadth and speed of identifying shocks and opportunities, supporting proactive planning; resource redundancy and alternative pathways reduce lock-in risks during industrial transition under shocks, accelerating the capture of new sustainable opportunities; cross-domain knowledge integration and competitive ecosystems drive green innovation, promoting resource flow toward efficient and clean sectors.

Finally, column (4) reports the regression results with Regulation as the moderating variable. The core interaction term *Economy_resil* × *Regulation* is highly significant and positive at the 1% level. These results support Hypothesis 4 proposed in this study, indicating that environmental regulation effectively enhances the positive impact of economic resilience on sustainable development. From the perspective of dynamic capability theory, environmental regulation strengthens the regional system’s ability to identify opportunities in green technologies and reconfigure its resource base by providing stable institutional expectations and clear transition directions. Under environmental regulatory constraints, regions are required not only to maintain economic stability but also to achieve sustainable development goals through innovative integration of production factors and cultivation of green industrial ecosystems. Institutional pressures compel regions to concentrate their resilient capacities on areas such as green technology innovation, industrial structure optimization, and improvements in environmental governance efficiency, thereby significantly deepening and broadening the contribution of economic resilience to sustainable development.

#### 4.2.2. Mediation effect.

Table 5 reports the regression results for Equations (3) and (4), the mediating effects models. Column (1) indicates that *Economy_resil1* significantly promotes *Market*. Column (2) shows that after incorporating *Market* into the regression, the main effect remains significant. The mediation test in Table 6 confirms a significant indirect effect, indicating that *Market* exerts a partial mediating role. Hypothesis 5 has been verified. This mediating pathway embodies the transformational logic of dynamic capabilities: economic resilience identifies market deficiencies and elevates marketization levels through institutional adjustments. Enhanced factor mobility reduces resource misallocation, directing factors toward green industries; environmental costs force green innovation and efficiency improvements. An optimized institutional environment stabilizes expectations for green investment, while competitive markets continuously incentivize sustainable innovation.

**Table 5:** Mediation effects.

	(1)	(2)
	*Market*	*SUS*
*Economy_resil1*	4.6851***	1.0071***
	(3.6843)	(17.0177)
*Market*		0.0072***
		(3.4704)
_cons	2.1756	0.6415***
	(0.5635)	(3.6181)
Control	YES	YES
Year FE	YES	YES
Province FE	YES	YES
*N*	544	544
adj. *R*^2^	0.7391	0.5956

**Table 6:** Test of mediating effect.

Mediating variable	Direct/Indirect effects	Coefficient	Standard deviation	Z value	P value	95% confidence interval
*Market*	Ind_eff	0.0058	0.0016	3.69	0.000	[0.0027 0.0089]
	Dir_eff	0.0072	0.0022	3.30	0.001	[0.0029 0.0115]

### 4.3. Heterogeneity analysis

Differences in regional development conditions have a significant heterogeneous impact on the effectiveness of economic resilience. To further explore this heterogeneity, this paper selects population density and industrial agglomeration as the first set of grouping variables, and external openness level and transportation infrastructure as the second set. The sample is divided into high and low-level groups based on the annual median of each variable [18]. Specifically, the first set of variables reflects the endogenous driving conditions of regional development, primarily characterizing the region’s internal economic structure and resource endowment. The second set of variables focuses on external connectivity and factor flow efficiency, measuring the region’s ability to interact with the external economic environment.

Regarding endogenous drivers of regional development, population density measures the spatial distribution characteristics of human resources, determining the agglomeration effect of economic activities and risk resistance capacity. Industrial agglomeration reflects the maturity of specialized division of labor and economies of scale, influencing the efficiency of supply chain coordination and recovery. Together, these factors form the self-reinforcing resilience foundation of a region: in areas with low population density, the economic system relies more on localized resource reorganization rather than scale effects, making resilience more significant in its marginal contribution to sustainable development. In contrast, regions with low industrial agglomeration, due to the lack of path dependence effects, are more likely to use resilience to promote structural transformation. Regarding external connectivity, the level of external openness reflects the depth of a region’s participation in the global value chain, which may dilute the local resilience’s focus on sustainable goals. Transportation infrastructure, on the other hand, represents the spatial penetration of physical capital and information flow, influencing the diffusion range of resilience benefits.

#### 4.3.1. Endogenous drivers.

The results in Table 7, columns (1) and (2), show that in regions with low population density, the positive effect of regional economic resilience is more pronounced, and this difference is confirmed through an inter-group coefficient test. This difference arises from the unique “resource reorganization advantage” in low-density areas: on the one hand, lower population pressure results in relatively looser resource constraints, such as land and environmental factors, providing more space for economic structural adjustment; on the other hand, these areas tend to rely more on localized production networks, and the improvement of economic resilience significantly optimizes the allocation efficiency of limited resources, generating greater marginal benefits.

**Table 7:** Heterogeneity analysis A.

	Population density		Industrial cluster	
	High	Low	High	Low
*Economy_resil1*	1.0565***	1.0872***	1.0538***	1.0800***
	(13.6406)	(11.2234)	(13.5828)	(11.1808)
_cons	0.6670**	0.4273**	0.6542**	0.4110**
	(2.1553)	(2.1109)	(2.1141)	(2.0450)
Control	YES	YES	YES	YES
Year FE	YES	YES	YES	YES
Province FE	YES	YES	YES	YES
*N*	279	265	279	265
adj. *R*^2^	0.6440	0.6656	0.6444	0.6686
Chow	5.05		4.28	
P value	0.0068		0.0143	

Table 7, columns (3) and (4), test the differences in industrial agglomeration. The results show that in regions with a more dispersed industrial distribution, economic resilience plays a more significant role. This difference is rooted in the “structural flexibility effect”: low-concentration areas avoid the lock-in risks caused by excessive industrial specialization, and their relatively diversified industrial base provides more adjustment options for responding to external shocks. Additionally, enterprises in industrially dispersed regions generally possess stronger cross-sector adaptability. When economic resilience improves, this adaptability can be more quickly translated into tangible outcomes for sustainable development.

#### 4.3.2. External connectivity.

External connectivity is also a key factor influencing the effectiveness of economic resilience. The results in Table 8, columns (1) and (2), confirm that in regions with lower levels of external openness, the enhancement of economic resilience leads to more noticeable improvements in sustainable development. This is because regions with limited external market access are more reliant on internal economic cycles. When economic resilience improves, it more effectively activates local industrial chain collaboration and optimizes regional resource allocation, resulting in greater development dividends. Additionally, the lower external dependence reduces the transmission of international market fluctuations to the local economy, making the effects of resilience-building easier to accumulate and consolidate within the region.

**Table 8:** Heterogeneity analysis B.

	Openness		Traffic construction	
	High	Low	High	Low
*Economy_resil1*	0.9656***	1.0318***	0.8223***	1.3401***
	(11.9115)	(11.7025)	(10.2461)	(11.8235)
_cons	0.4281	−0.0145	0.6434**	0.4647*
	(1.4800)	(−0.0760)	(2.1883)	(1.9681)
Control	YES	YES	YES	YES
Year FE	YES	YES	YES	YES
Province FE	YES	YES	YES	YES
*N*	279	265	279	265
adj. *R*^2^	0.4907	0.7777	0.6750	0.4701
Chow	5.56		6.23	
P value	0.0041		0.0021	

Table 8, columns (3) and (4), illustrate the impact of economic resilience under differing levels of transportation infrastructure. The results show that the effectiveness of economic resilience varies depending on the level of infrastructure. This phenomenon can be explained by the “local focus effect”: limited transportation conditions objectively slow the outflow of factors, forcing regions to enhance internal economic resilience to address development challenges. These constraints prompt local governments and enterprises to place greater emphasis on building long-term mechanisms, such as cultivating local innovation ecosystems and improving industrial chain support. In contrast, regions with high transportation convenience, while able to access external resources more easily, also face risks such as the outflow of high-quality factors and industrial “hollowing,” which partially weakens the long-term benefits of economic resilience.

### 4.4. Robustness check

This study addresses potential endogeneity issues by lagging both the independent and dependent variables. Table 9 presents the regression results. In column (1), the independent variable is lagged by one period, resulting in *L Economy_resil1*. In column (2), the dependent variable is lagged by one period, resulting in *L.SUS*. Both regressions show significant positive results at the 1% level, indicating that the effect of Economy_resil1 on *SUS* is free from endogeneity interference. In column (3), *Economy_resil1* is replaced with *Economy_resil2*, Column (4) substitutes *SUS* with *SUS1*, Column (5) excludes samples from 2020 onward affected by the pandemic following Choudhary, Dal Barco [19] and Gormsen and Koijen [20]. Coefficients remain significant across all regressions, again validating Hypothesis 1.

**Table 9:** Robustness check.

	(1)	(2)	(3)	(4)	(5)
	*SUS*	*L.SUS*	*SUS1*	*SUS*	*SUS*
*L.Economy_resil1*	0.8347***				
	(11.6424)				
*Economy_resil1*		0.9156***	7.6992***		0.9624***
		(14.3304)	(9.6312)		(13.6358)
*Economy_resil2*				0.3190***	
				(6.0113)	
_cons	0.6566***	0.7778***	−7.1458***	0.3628	0.6441***
	(2.9144)	(3.7644)	(−2.9441)	(1.6440)	(3.5161)
Control	YES	YES	YES	YES	YES
Year FE	YES	YES	YES	YES	YES
Province FE	YES	YES	YES	YES	YES
*N*	512	512	544	544	482
adj. *R*^2^	0.4643	0.5461	0.9128	0.3696	0.5323

## 5. Discussion

### 5.1. Result and discussion

The findings of this study present substantial contrasts with existing literature. First, based on endogenous growth theory, Hypothesis 1 was proposed and validated. This discovery aligns with the positive effect of resilience on long-term development observed by Bristow and and Healy [21] in European contexts. Second, this research explores the mechanisms through which economic resilience operates, verifying Hypotheses 2 and 3. Hypothesis 2 confirms that human capital positively moderates the relationship between economic resilience and sustainable development. This supports the skills-driven resilience theory proposed by Kakderi and and Tasopoulou [22], where skilled labor not only enhances productivity but also enables economic systems to dynamically adjust knowledge absorption strategies, maintaining adaptability during technological transitions. Hypothesis 3 confirms that industrial diversity strengthens the efficacy of economic resilience, echoing Boschma [4] complex adaptive systems theory.

Hypothesis 4 validates the amplifying effect of environmental regulation on regional economic resilience, which aligns with Li, Xu [23]’s finding that the digital economy promotes green economic growth while environmental regulations positively influence this development. This study further deepens this understanding from a dynamic capabilities perspective, revealing that environmental regulation guides regions to concentrate their resilient capacities in the fields of green innovation and structural transformation by providing clear green technology expectations and stable institutional signals, thereby significantly enhancing the efficacy of economic resilience in the dimension of sustainable development.

Hypothesis 5 verifies the pathway through which economic resilience operates. The results confirm that enhancing regional marketization levels constitutes an important mechanism through which economic resilience promotes sustainable development. Compared with Liu, T.L. [24] ’s research, this study extends the understanding of marketization’s impact on green economic development by identifying its mediating role in the resilience-sustainability relationship and elucidating the specific mechanisms through which market-based resource allocation translates resilient capacities into sustainable outcomes. Finally, heterogeneity analysis reveals how regional development disparities influence economic resilience outcomes. It is worth noting that our data indicates economic resilience’s promoting effect on sustainable development is more pronounced in regions with low openness. This finding diverges from Donaldson and Hornbeck [23] external resource dependence theory. We argue that a possible explanation is that for economies at specific development stages, excessively rapid and deep openness may inhibit the cultivation of local innovation ecosystems, while moderate openness may provide necessary buffer and learning space for accumulating indigenous capabilities, thereby more fully unleashing economic resilience’s efficacy. This nuance extends Donaldson’s (2016) market access approach [23] by incorporating resilience in less open regions.

### 5.2. Theoretical implication

This study addresses the limitations in existing literature regarding the narrow connotation of resilience—often limited to short-term recovery metrics without considering long-term adaptive transformations (Sensier et al., 2016; Boschma, 2015)—and ambiguous mechanisms of action, such as unclear pathways linking resilience to broader outcomes (Martin, 2012), focusing on how regional economic resilience promotes sustainable development.

Theoretical advancements are achieved primarily in the following aspects: First, it extends the temporal dimension and theoretical connotation of economic resilience research by integrating evolutionary perspectives on path dependency and systemic adaptation (Boschma, 2015). By constructing a comprehensive and robust framework for measuring economic resilience—drawing on composite indices that encompass resistance, adaptability, and innovation (Downing et al., 2018; Sensier et al., 2016)—and empirically revealing the sustained promoting effect of economic resilience on sustainable development using long-term panel data, this study clarifies the mediating pathway of marketization level (Fu et al., 2025).

This enriches the theoretical explanation of how resilience transforms into developmental momentum at the mechanistic level, advancing market systems resilience frameworks that emphasize resource allocation efficiency and institutional adjustments in developing contexts (Downing et al., 2018). Second, departing from traditional approaches examining single moderating variables (e.g., Frenken et al., 2007), this paper develops a synergistic moderating model integrating human capital and industrial diversity. It uncovers the compound mechanisms through which capability enhancement and structural synergy amplify resilience effects—such as how human capital enables flexible resource reallocation amid diversification (He et al., 2022; Kakderi and Tasopoulou, 2017)—providing theoretical underpinnings for understanding adaptive transformation driven by multi-element interactions within regional systems.

### 5.3. Policy recommendations

Based on the aforementioned research findings, we propose the following policy considerations with enhanced heuristic value and contextual adaptability, aiming to provide reference for regional development practices: For less developed regions with lower agglomeration levels, given the critical role of human capital and industrial diversity identified in this study, policies should prioritize investment in skills enhancement programs and promote synergies among local industries to address innovation challenges stemming from geographical dispersion. Provincial authorities may explore establishing guiding funds to support such collaborations. For regions with lower openness levels and relatively weak infrastructure, the advancement of market-oriented reforms and openness requires prudent assessment of implementation pace and supporting measures. Policymakers should monitor dynamic changes in regional industrial structures. Establishing early-warning mechanisms helps identify risks associated with excessive concentration in single industries, enabling timely guidance to support SME development and diversified investment.

When evaluating regional development performance, it is recommended to incorporate considerations regarding the efficiency with which economic resilience translates into tangible development outcomes—rather than focusing solely on input scale—to better direct resources toward resilience-building initiatives that yield substantive benefits. While promoting factor mobility to enhance efficiency, supporting mechanisms should be designed to mitigate potential exacerbation of regional development disparities and ensure shared developmental outcomes. Based on this, further considerations can be made by combining the research conclusions with the following aspects: When promoting environmental regulatory policies, it is possible to explore the combination of these policies with industrial diversification and human capital investment, and formulate a comprehensive policy package. However, it is necessary to pay attention to the actual constraints on the implementation of environmental regulations in different regions and adopt differentiated promotion strategies.

During the process of marketization, it is necessary to focus on cultivating the capabilities of local market entities, especially in regions with weak external connectivity, and consider enhancing their adaptability to market reforms by improving the business environment and reducing institutional transaction costs. It is recommended to strengthen the construction of a regional economic resilience monitoring and evaluation system, regularly tracking changes in mechanism variables such as industrial structure diversity, human capital reserves, and environmental regulation intensity, to provide a basis for policy adjustments. It must be emphasized that the effectiveness of any policy intervention is highly dependent on specific regional institutional environments, development stages, resource endowments, and policy implementation capacities. Thus, policies should be tailored, drawing on the heterogeneous effects identified, to maximize resilience’s role in sustainability. The insights provided by this study aim to highlight potential pathways for enhancing economic resilience to promote sustainable development across different regional contexts, along with key factors requiring attention. Policymakers should conduct prudent assessments of local realities, policy space, and feasibility to implement contextually adapted adjustments.

### 5.4. Limitations

Although this study attempted to explore the relationship between economic resilience and sustainable development as well as its mechanisms, it still has several significant limitations that need to be overcome in future research. Firstly, this study mainly relied on provincial-level data for analysis. This scale selection has advantages in data comparability, but it also has significant limitations. The aggregated data at the provincial level is difficult to capture the significant differences in recovery paths among different cities, counties, and urban-rural areas. To address these limitations, future research can actively promote the collection and analysis of data at the municipal and county levels, as well as even more micro levels. Spatial econometric methods at this level will be highly valuable, as they can effectively depict the spatial dependence and heterogeneity of economic resilience and sustainable development, and more accurately identify the impacts of local shocks. The application of machine learning technology to assist in analysis can help handle high-dimensional data, identify complex nonlinear relationships and interactions between variables, and possibly discover resilience driving patterns and threshold effects that traditional econometric methods may struggle to capture, thereby providing a deeper understanding of how factors such as human capital, industrial diversity, institutions, and social capital interweave to influence sustainable development.

## 6. Conclusion

This paper uses data from all provinces in China during the period from 2004 to 2023 to construct a two-way fixed effects model for empirical research, and has reached the following conclusions: First, it is confirmed that regional economic resilience positively impacts sustainable development, even after replacing variables, changing the model, and reducing the sample size, the conclusion remains robust. Second, the study explored the relevant mechanisms that influence the effectiveness of economic resilience, and found that as industrial diversity increases, human capital capabilities improve, and environmental regulations become more stringent, the positive effects of regional economic resilience have been strengthened. Third, the study validates that enhancing the level of marketization facilitates regional sustainable development through economic resilience. Finally, based on the results of the heterogeneity analysis, the differences in endogenous drivers and external connectivity conditions have varied impacts on the positive influence of regional economic resilience.

## Supporting information

S1 Appendix Table A.1. *SUS* indicator system. Table A.2. *Economy resil1* indicator system. Table A.3. *Economy resil2* indicator system.
(DOCX)
