# Supplementary material for: Regional economic resilience and sustainable development: Does industrial diversity matter?
Source: PLoS One. 2026 Feb 13;21(2):e0342488. doi: 10.1371/journal.pone.0342488 (PMC12904456; doi:10.1371/journal.pone.0342488)
Supplement: S1 Appendix — Table A.1. SUS indicator system. Table A.2. Economy resil1 indicator system. Table A.3. Economy resil2 indicator system. (DOCX) [file pone.0342488.s001.docx]

**Appendix A.1 *SUS* indicator system**

**Table A.1. *SUS* indicator system**

| Primary indicator | Secondary indicator | Tertiary indicator |
| --- | --- | --- |
| Sustainable development | Economic | Local government general budget revenue |
|  |  | Total retail sales of consumer goods |
|  |  | Local government general budget expenditure (billion yuan) |
|  | Environmental | Forest coverage rate |
|  |  | Per capita Park green space area |
|  |  | Daily urban sewage treatment capacity |
|  |  | Municipal solid waste harmless disposal rate |
|  | Social | Urban disposable Income per capita |
|  |  | Number of beds in health institutions |
|  |  | Public transportation vehicles per 10000 people |
|  |  | Urban registered unemployment rate |

**Appendix A.2 *Economy_resil1* indicator system**

**Table A.2. *Economy_resil1* indicator system**

| Primary indicator | Criteria layer | Sub-criteria layer | Indicator layer | Polarity |
| --- | --- | --- | --- | --- |
| Regional economic resilience | Resistance and recovery capacity | Financial development | Value added of financial industry | + |
|  |  | Trade pressure | Foreign trade dependence | - |
|  |  | Urban-rural structure | Urban-rural income ratio | - |
|  |  | Unemployment relief | Number of unemployment insurance participants | + |
|  |  | Social assistance | Cumulative balance of basic pension insurance | + |
|  |  | Economic foundation | Regional gross domestic product | + |
|  | Adaptation and adjustment capacity | Market potential | Total retail sales of consumer goods | + |
|  |  | Industrialization development | Industrial value added (billion yuan)/ Regional GDP | + |
|  | Innovation and evolution capacity | Innovation output | Number of patents granted | + |

**Appendix A.3 *Economy_resil2* indicator system**

**Table A.3. *Economy_resil2* indicator system**

| Primary indicator | Secondary indicator | Tertiary indicator |
| --- | --- | --- |
| Regional economic resilience | Resistance and recovery capacity | Regional gross domestic product |
|  |  | Urban disposable income per capita |
|  |  | Rural disposable income per capita |
|  |  | Total import and export volume |
|  |  | Urban registered unemployment rate |
|  | Adaptation and adjustment capacity | Local government fiscal revenue |
|  |  | Local government fiscal expenditure |
|  |  | Per capita total retail sales of consumer goods |
|  |  | Fixed asset investment |
|  | Innovation and evolution capacity | Number of granted domestic invention patents |
|  |  | Local government expenditure on education |
|  |  | Local government expenditure on science and technology |
|  |  | Industrial structure advancement |
